# Supplementary material for: Response surface methodology for the mixed fungal fermentation of Codonopsis pilosula straw using Trichoderma reesei and Coprinus comatus
Source: PeerJ. 2023 Aug 14;11:e15757. doi: 10.7717/peerj.15757 (PMC10434135; doi:10.7717/peerj.15757)
Supplement: Supplemental Information 2 [file peerj-11-15757-s002.docx]

**Table 2** Response surface design factor levels and coding

| **Levels** | **Factors** | | | |
| --- | --- | --- | --- | --- |
|  | **Fungus ratio  (A)** | **Fungal inoculum(B) /%** | **Additive amount of corn flour  (C) /%** | **Fermentation time (D) /d** |
| -1 | 2:8 | 8 | 8 | 13 |
| 0 | 3: 7 | 10 | 10 | 15 |
| 1 | 4: 6 | 12 | 12 | 17 |
