# Supplementary material for: Response surface methodology for the mixed fungal fermentation of Codonopsis pilosula straw using Trichoderma reesei and Coprinus comatus
Source: PeerJ. 2023 Aug 14;11:e15757. doi: 10.7717/peerj.15757 (PMC10434135; doi:10.7717/peerj.15757)
Supplement: Supplemental Information 3 [file peerj-11-15757-s003.docx]

**Table 3** Box-Behnken response surface design and results

| **Experimental** | **Factors^1^** | | | | **CDR^2^ (%)** | **LDR^3^ (%)** |
| --- | --- | --- | --- | --- | --- | --- |
| **No．** | **A** | **B** | **C** | **D** |  |  |
| 1 | -1 | -1 | 0 | 0 | 8.84 | 8.4 |
| 2 | 1 | -1 | 0 | 0 | 14.15 | 10.84 |
| 3 | -1 | 1 | 0 | 0 | 10.01 | 11.62 |
| 4 | 1 | 1 | 0 | 0 | 7.76 | 6.45 |
| 5 | 0 | 0 | -1 | -1 | 9.73 | 5.57 |
| 6 | 0 | 0 | 1 | -1 | 8.24 | 5.95 |
| 7 | 0 | 0 | -1 | 1 | 8.04 | 6.05 |
| 8 | 0 | 0 | 1 | 1 | 12.82 | 8.79 |
| 9 | -1 | 0 | 0 | -1 | 9.47 | 8.04 |
| 10 | 1 | 0 | 0 | -1 | 10.12 | 7.12 |
| 11 | -1 | 0 | 0 | 1 | 9.47 | 10.04 |
| 12 | 1 | 0 | 0 | 1 | 13.89 | 10.04 |
| 13 | 0 | -1 | -1 | 0 | 11.52 | 9.54 |
| 14 | 0 | 1 | -1 | 0 | 9.06 | 4.6 |
| 15 | 0 | -1 | 1 | 0 | 11.11 | 8.65 |
| 16 | 0 | 1 | 1 | 0 | 9.79 | 8.44 |
| 17 | -1 | 0 | -1 | 0 | 9.43 | 8.11 |
| 18 | 1 | 0 | -1 | 0 | 8.95 | 6.86 |
| 19 | -1 | 0 | 1 | 0 | 9.02 | 10.37 |
| 20 | 1 | 0 | 1 | 0 | 13.02 | 7.77 |
| 21 | 0 | -1 | 0 | -1 | 9.99 | 8.52 |
| 22 | 0 | 1 | 0 | -1 | 8.35 | 5.67 |
| 23 | 0 | -1 | 0 | 1 | 12.09 | 9.07 |
| 24 | 0 | 1 | 0 | 1 | 9.82 | 6.82 |
| 25 | 0 | 0 | 0 | 0 | 11.41 | 9.87 |
| 26 | 0 | 0 | 0 | 0 | 13.94 | 10.13 |
| 27 | 0 | 0 | 0 | 0 | 13.09 | 10.28 |
| 28 | 0 | 0 | 0 | 0 | 12.78 | 11.03 |
| 29 | 0 | 0 | 0 | 0 | 12.78 | 10.99 |

^1^A, fungus ratios; B, fungal fermentation inoculation amount; C, additive amount of corn flour; D, fermentation time. ^2^CDR, cellulose degradation rate; ^3^LDR, lignin degradation rate.
