# Supplementary material for: Response surface methodology for the mixed fungal fermentation of Codonopsis pilosula straw using Trichoderma reesei and Coprinus comatus
Source: PeerJ. 2023 Aug 14;11:e15757. doi: 10.7717/peerj.15757 (PMC10434135; doi:10.7717/peerj.15757)
Supplement: Supplemental Information 4 [file peerj-11-15757-s004.docx]

**Table 4** Variance analysis of cellulose degradation rate response surface results

| **Source^1^** | **Sum of** | **df^2^** | **Mean** | ***F*-value** | ***p*-value** |
| --- | --- | --- | --- | --- | --- |
|  | **Squares** |  | **Square** |  |  |
| Model | 100.2 | 14 | 7.16 | 13.19 | < 0.0001 |
| A-Fungus ratios | 11.25 | 1 | 11.25 | 20.73 | 0.0005 |
| B-Fungal fermentation  inoculation amount | 13.88 | 1 | 18.52 | 25.58 | 0.0002 |
| C-Additive amount  of corn flour | 4.39 | 1 | 4.39 | 8.09 | 0.013 |
| D-Fermentation time | 8.72 | 1 | 8.72 | 16.07 | 0.0013 |
| AB | 14.31 | 1 | 14.31 | 26.36 | 0.0002 |
| AC | 5.01 | 1 | 5.01 | 9.24 | 0.0088 |
| AD | 3.35 | 1 | 3.35 | 6.53 | 0.0288 |
| BC | 0.33 | 1 | 0.33 | 0.61 | 0.4489 |
| BD | 0.097 | 1 | 0.097 | 0.18 | 0.679 |
| CD | 9.85 | 1 | 9.85 | 18.15 | 0.0008 |
| A^2^ | 7.58 | 1 | 7.58 | 13.96 | 0.0022 |
| B^2^ | 10.69 | 1 | 10.69 | 19.71 | 0.0006 |
| C^2^ | 14.67 | 1 | 14.67 | 27.04 | 0.0001 |
| D^2^ | 11.67 | 1 | 11.67 | 21.5 | 0.0004 |
| Residual | 7.6 | 14 | 0.54 |  |  |
| Lack of Fit | 4.28 | 10 | 0.43 | 0.52 | 0.8196 |
| Pure Error | 3.32 | 4 | 0.83 |  |  |
| Cor Total | 107.8 | 28 |  |  |  |
| R^2^ | 0.93 | R_Adj_^2^ | 0.86 |  |  |

^1^A, fungus ratios; B, fungal fermentation inoculation amount; C, additive amount of corn flour; D, fermentation time; AB-CD, Means the interaction of two factors; A^2^-D^2^, Means quadratic term; R^2^, correlation coefficient. ^2^R_Adj_^2^, adjusted coefficient of determination;
