# Supplementary material for: Response surface methodology for the mixed fungal fermentation of Codonopsis pilosula straw using Trichoderma reesei and Coprinus comatus
Source: PeerJ. 2023 Aug 14;11:e15757. doi: 10.7717/peerj.15757 (PMC10434135; doi:10.7717/peerj.15757)
Supplement: Supplemental Information 5 [file peerj-11-15757-s005.docx]

**Table 5** Variance analysis of lignin degradation rate response surface results

| **Source^1^** | **Sum of** | **df** | **Mean** | ***F*-value** | ***p*-value** |
| --- | --- | --- | --- | --- | --- |
|  | **Squares** |  | **Square** |  |  |
| Model | 95.16 | 14 | 6.80 | 13.78 | < 0.0001 |
| A-Fungus ratios | 4.69 | 1 | 4.69 | 9.51 | 0.0081 |
| B-Fungal fermentation  inoculation amount | 10.85 | 1 | 10.85 | 21.98 | 0.0003 |
| C-Additive amount  of corn flour | 7.12 | 1 | 7.12 | 14.42 | 0.002 |
| D-Fermentation time | 8.25 | 1 | 8.25 | 16.73 | 0.0011 |
| AB | 14.47 | 1 | 14.47 | 29.32 | < 0.0001 |
| AC | 0.46 | 1 | 0.46 | 0.93 | 0.3519 |
| AD | 0.21 | 1 | 0.21 | 0.43 | 0.5235 |
| BC | 5.57 | 1 | 5.57 | 11.29 | 0.0047 |
| BD | 0.089 | 1 | 0.089 | 0.18 | 0.6779 |
| CD | 1.4 | 1 | 1.4 | 2.85 | 0.1138 |
| A^2^ | 0.039 | 1 | 0.039 | 0.079 | 0.7829 |
| B^2^ | 5.95 | 1 | 5.95 | 12.06 | 0.0037 |
| C^2^ | 24.57 | 1 | 24.57 | 49.8 | < 0.0001 |
| D^2^ | 21.64 | 1 | 21.64 | 43.85 | < 0.0001 |
| Residual | 6.91 | 14 | 0.49 |  |  |
| Lack of Fit | 5.82 | 10 | 0.58 | 2.13 | 0.2428 |
| Pure Error | 1.09 | 4 | 0.27 |  |  |
| Cor Total | 102.07 | 28 |  |  |  |
| R^2^ | 0.93 | R_Adj_^2^ | 0.86 |  |  |

^1^A, fungus ratios; B, fungal fermentation inoculation amount; C, additive amount of corn flour; D, fermentation time; AB-CD, Means the interaction of two factors; A^2^-D^2^, Means quadratic term; R^2^, correlation coefficient. ^2^R_Adj_^2^, adjusted coefficient of determination;
