# Supplementary figures and images for: Response surface methodology for the mixed fungal fermentation of Codonopsis pilosula straw using Trichoderma reesei and Coprinus comatus
Source: PeerJ. 2023 Aug 14;11:e15757. doi: 10.7717/peerj.15757 (PMC10434135; doi:10.7717/peerj.15757)

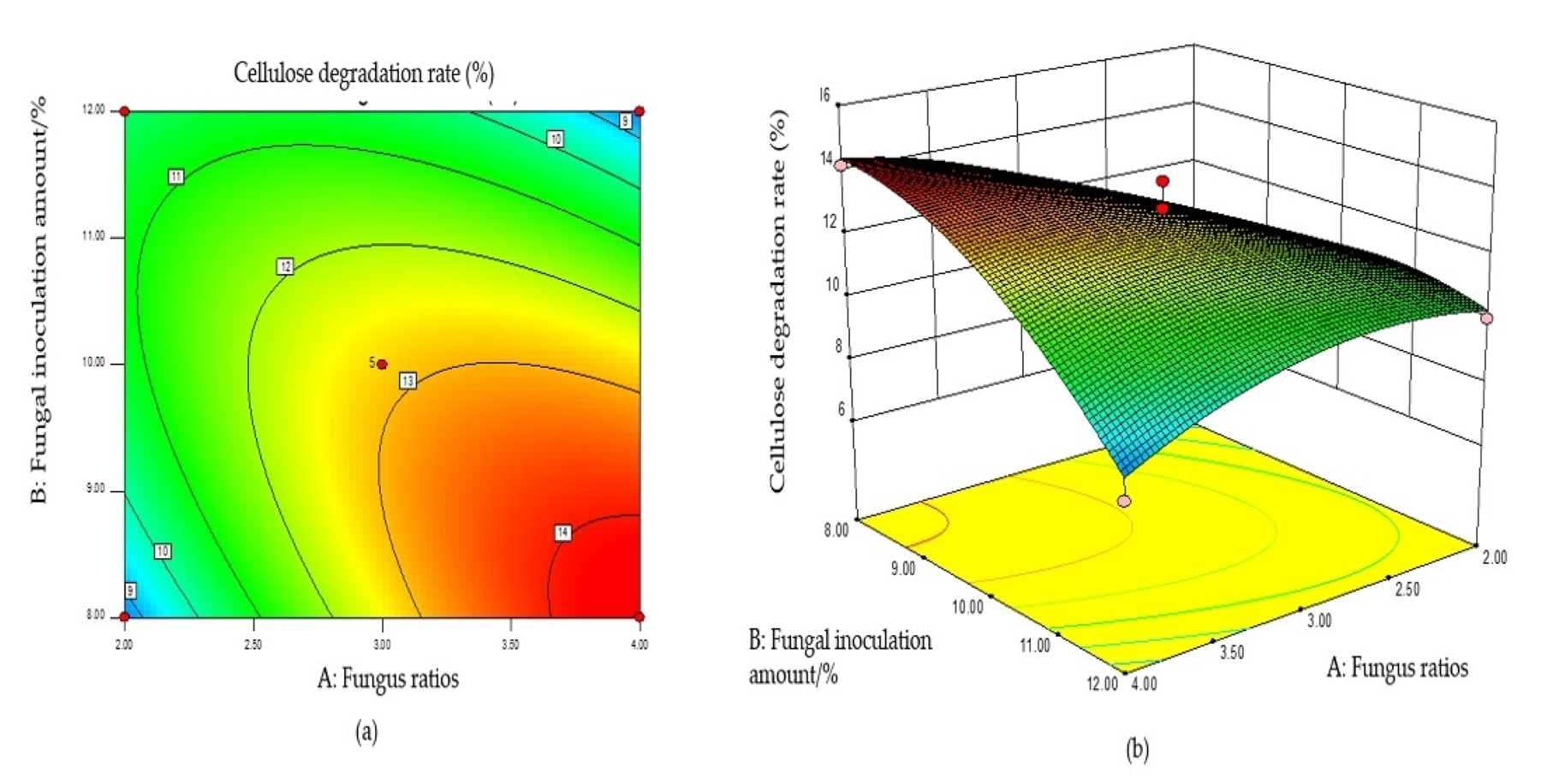

Supplement: Supplemental Information 6 [file peerj-11-15757-s006.jpg]

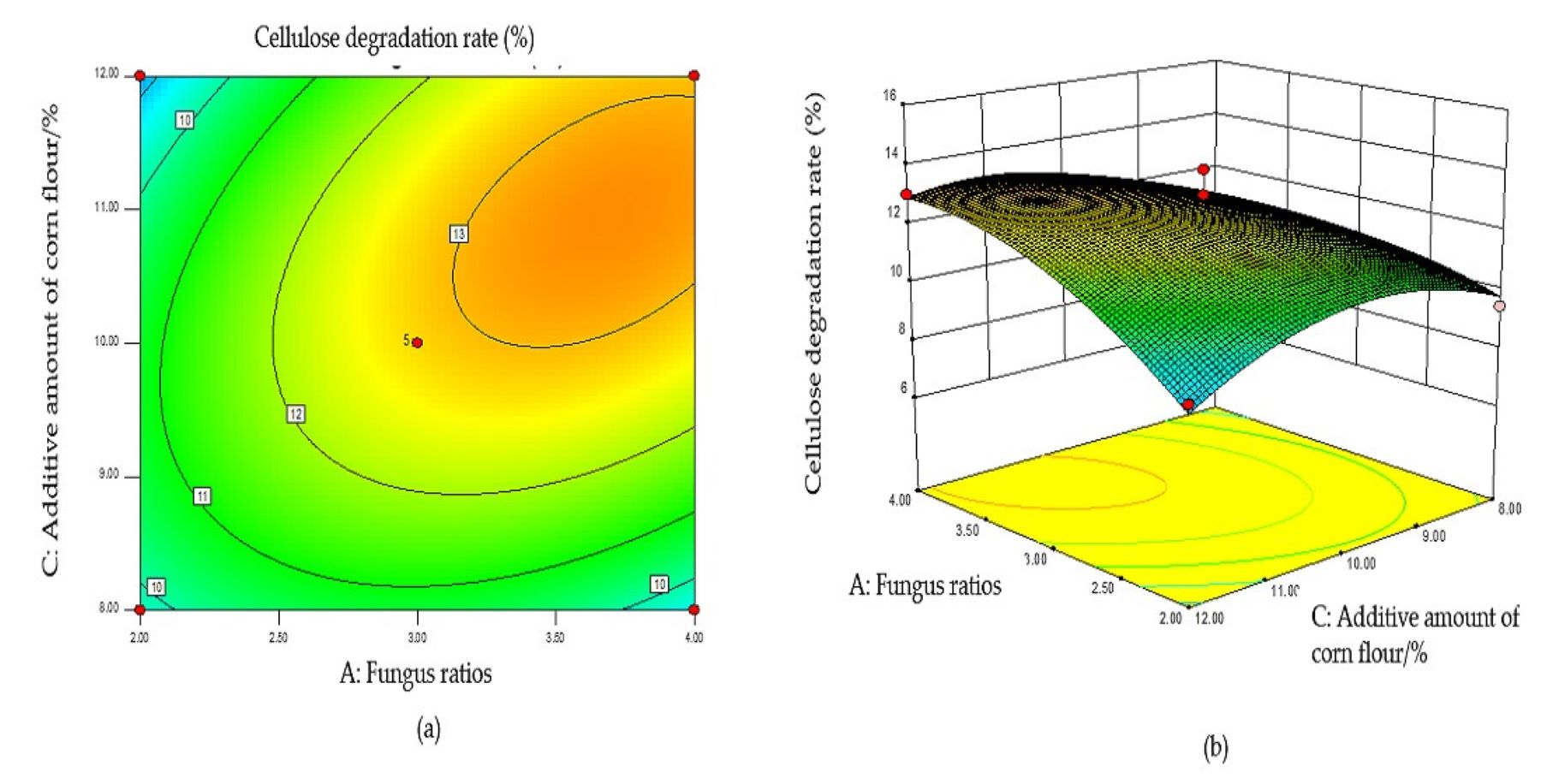

Supplement: Supplemental Information 7 [file peerj-11-15757-s007.jpg]

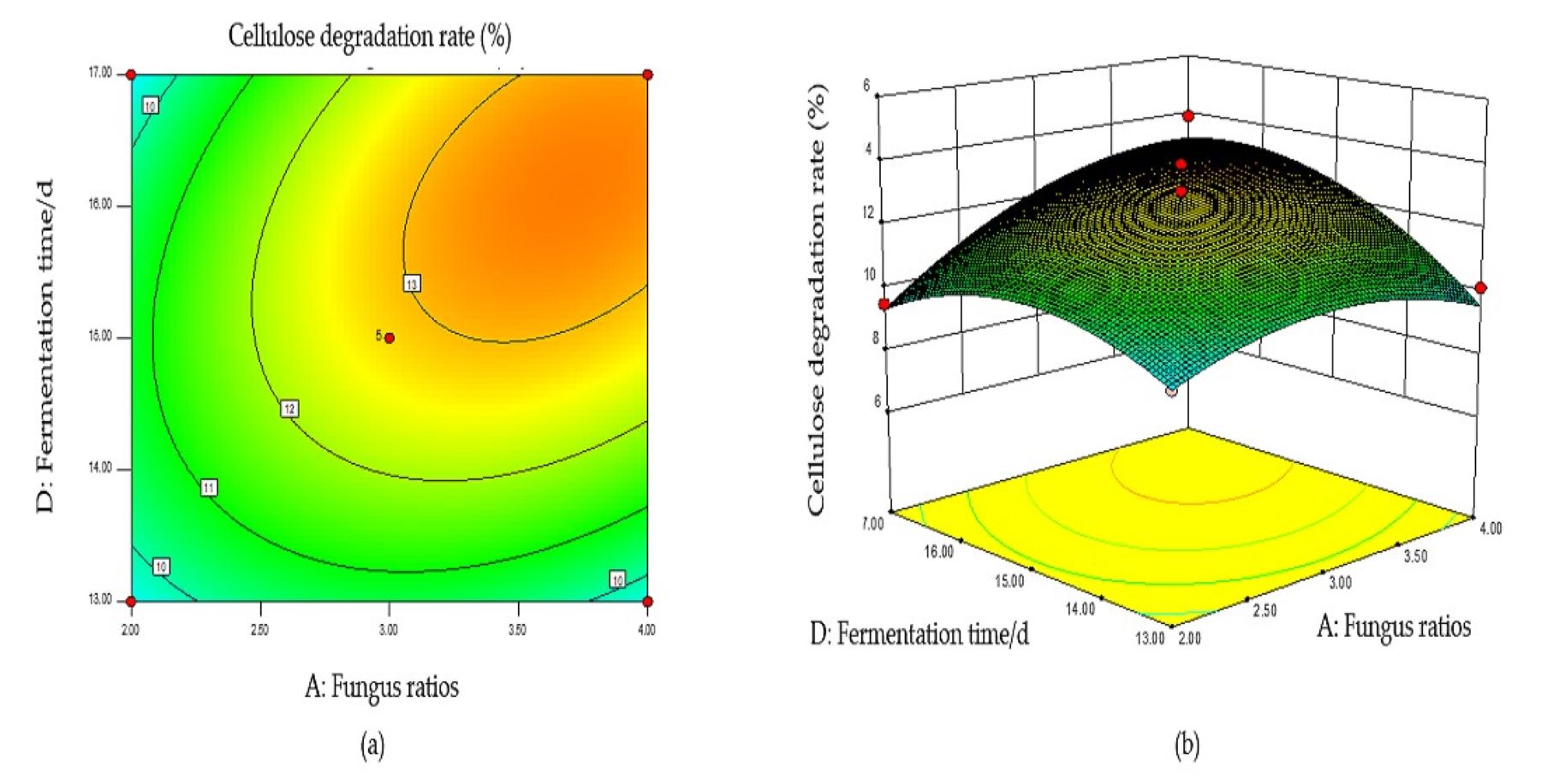

Supplement: Supplemental Information 8 [file peerj-11-15757-s008.jpg]

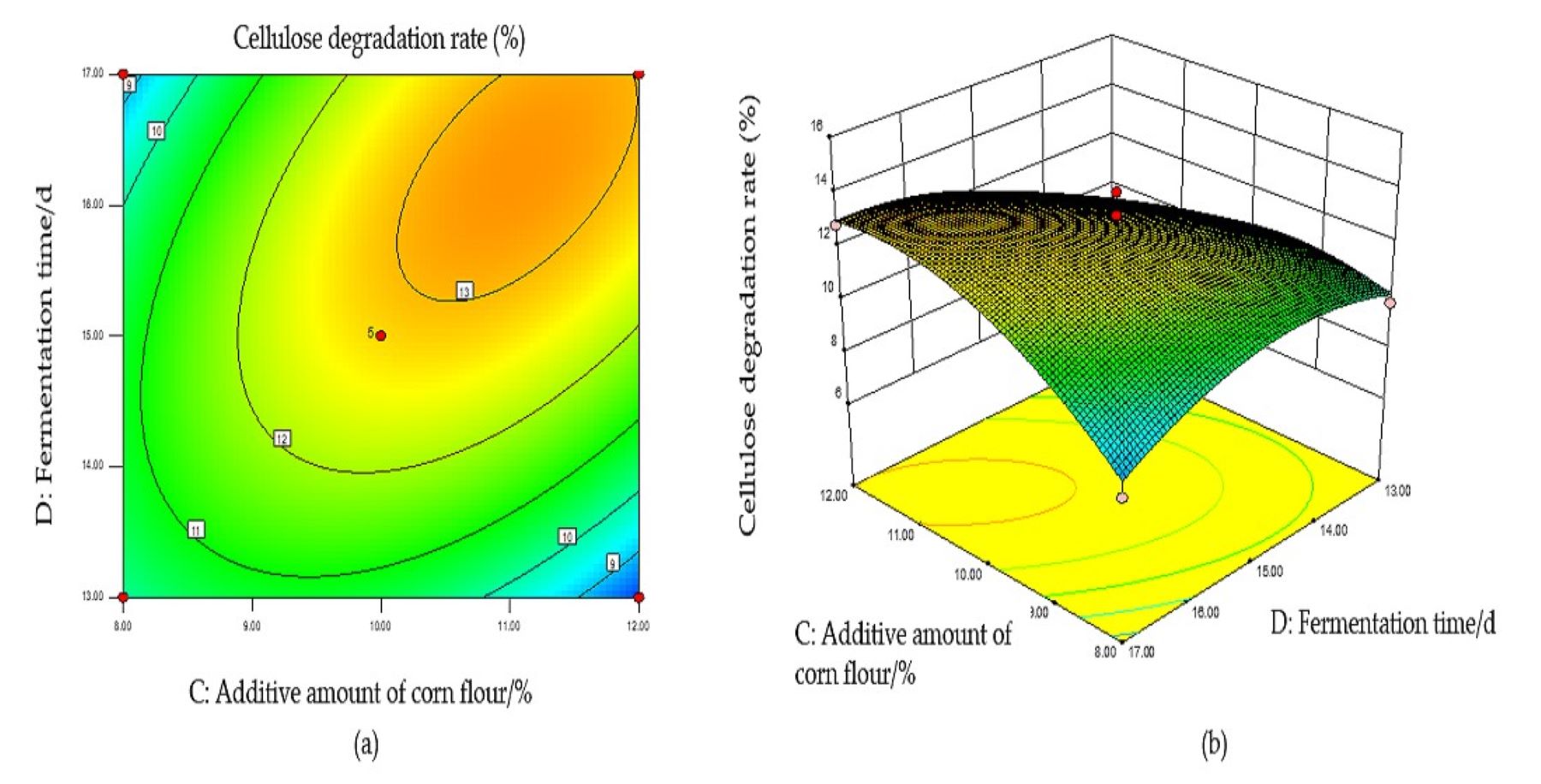

Supplement: Supplemental Information 9 [file peerj-11-15757-s009.jpg]

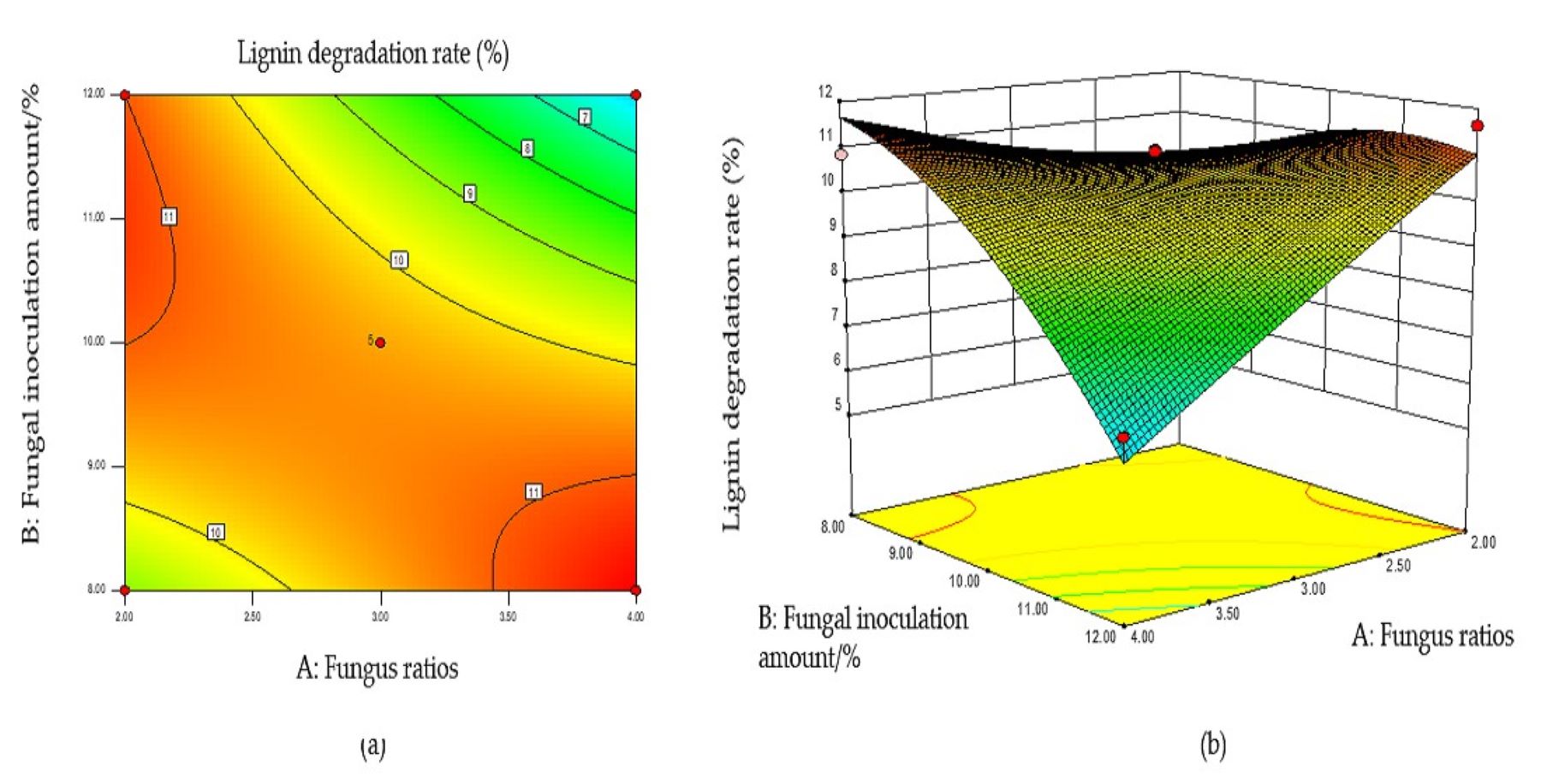

Supplement: Supplemental Information 10 [file peerj-11-15757-s010.jpg]

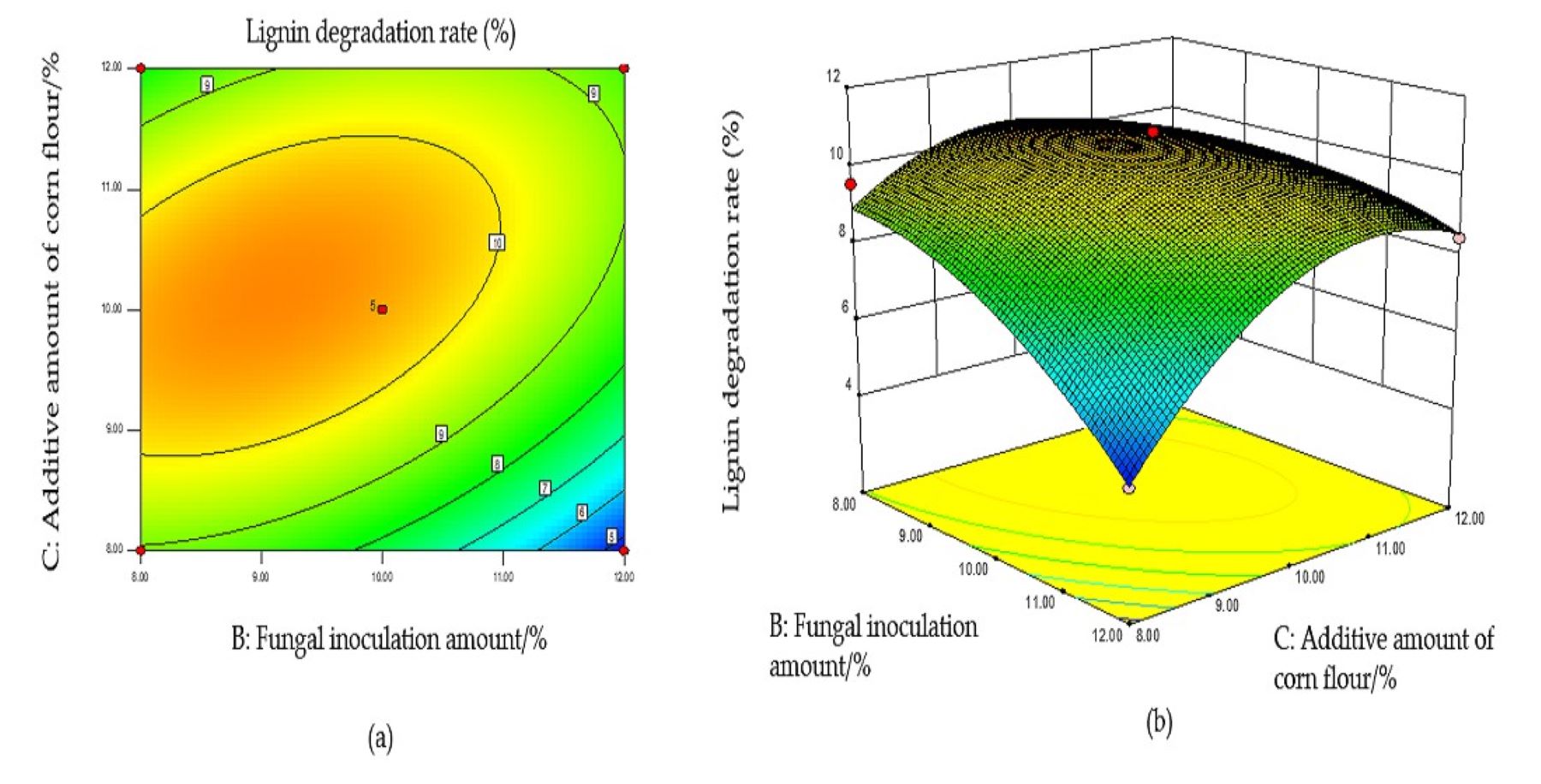

Supplement: Supplemental Information 11 [file peerj-11-15757-s011.jpg]
